# Women Entrepreneurs and Innovation Strategies: Driving Inclusive Fintech Business Growth in Sub-Saharan Africa

**DOI:** 10.12688/f1000research.167438.1

**Published:** 2025-08-26

**Authors:** Tom Ongesa Nyamboga

**Affiliations:** 1 BUSINESS ADMINISTRATION, Kampala International University - Western Campus, Bushenyi, Western Region, 71, Uganda

**Keywords:** Empowering Innovation, Women Entrepreneurs, Fintech Business Growth, Sub-Saharan Africa

## Abstract

This review examines how women entrepreneurs can drive financial technology (fintech) business growth in Sub-Saharan Africa. While fintech is expanding rapidly across the region, the unique role of women-led ventures in promoting innovation and inclusive growth has been underexplored. A narrative literature review of studies from 2015 to 2025 was conducted, with qualitative findings analyzed thematically. Results show that women entrepreneurs leverage mobile technology, AI, blockchain, digital payments, and innovation hub collaborations to enhance market access, financial inclusion, and capacity building. Nonetheless, persistent challenges such as limited digital literacy, regulatory barriers, gender biases, and a lack of gender-disaggregated data constrain sustainable growth and adoption of women-led fintech ventures. These findings highlight the need for targeted policy measures to improve women’s access to finance, technology, and networks, strengthening inclusive fintech ecosystems across Sub-Saharan Africa. Overall, empowering women entrepreneurs is critical for fostering equitable and sustainable fintech innovation in the region.

## Introduction

The intersection of inclusive fintech business growth and women’s innovation highlights how women entrepreneurs are using technology to solve financial access challenges ( Liang et al., 2024; Irwin). By developing digital platforms and tailored financial services, they expand opportunities for underserved groups ( Adelaja et al., 2024). This approach strengthens women’s roles in the digital economy while promoting inclusive innovation and long-term fintech growth ( Liang et al., 2024; Jam’an, 2024).

Women entrepreneurs significantly contribute to fintech growth in Sub-Saharan Africa, where financial inclusion remains a major challenge ( Kedir & Kouame, 2022). Fintech, which involves technology-driven financial services, is transforming how financial products are accessed and delivered by improving efficiency and affordability ( Josyula & Expert, 2021; Malhotra & Malhotra, 2023). These entrepreneurs bring innovative solutions, such as mobile money platforms, AI-based credit scoring, and blockchain transactions, that address the needs of underserved populations ( Poon et al., 2024). However, women-led fintech ventures face obstacles like limited capital access, gender biases, regulatory hurdles, and insufficient technical training ( Omiwale, 2024; Chakravarty et al., 2025; Irwin et al., 2025). This study examines how women leverage innovation to overcome these challenges, aiming to inform policies that support their success and expand inclusive fintech ecosystem.

Current literature highlights the rapid growth of fintech in Sub-Saharan Africa, fueled by widespread mobile technology use, rising demand for financial inclusion, and innovations like mobile money, AI-driven credit scoring, and blockchain ( Mhlanga, 2024; Daudu et al., 2025). Fintech effectively addresses gaps in traditional banking, especially for underserved rural and informal populations ( Adelaja et al., 2024; Kamal et al., 2025). Research shows women entrepreneurs creatively utilize fintech platforms to develop community savings and informal credit schemes targeting low-income groups ( Omowole et al., 2024; Ndandani, 2022). However, existing studies often overlook women’s specific roles, innovation strategies, and challenges in fintech growth. This review aims to bridge this gap by analyzing how women-led fintech innovations support scalable business growth and sustainable development, offering policy and investment recommendations for inclusive fintech ecosystems.

Based on the review, the objectives of this study would be:

• To examine how women entrepreneurs in Sub-Saharan Africa leverage fintech innovations to drive business growth and financial inclusion.
• To identify the unique challenges and barriers faced by women-led fintech enterprises in scaling their businesses.
• To analyze the specific innovation strategies employed by women entrepreneurs within the fintech ecosystem that contribute to sustainable and scalable business expansion.
• To explore the role of gender dynamics in shaping fintech innovation and business development in the region.
• To provide actionable recommendations for policymakers, investors, and stakeholders to support and enhance women’s participation and success in the fintech sector, fostering inclusive and gender-responsive fintech ecosystems.

## Materials and methods

This narrative review adopted a qualitative methodology guided by thematic analysis to explore how women entrepreneurs in Sub-Saharan Africa can drive fintech business growth. The approach enabled a structured examination of patterns, concepts, and emerging trends across five thematic areas: mobile technology, artificial intelligence, blockchain, digital payment integration, and collaboration with innovation hubs and incubators. By synthesizing evidence from academic literature, policy reports, and case studies, the methodology provided a comprehensive understanding of the opportunities and challenges women face in leveraging fintech innovations. Thematic analysis offered flexibility for identifying both predefined and emerging insights relevant to the research focus ( Braun & Clarke, 2006).

### Data collection methods

This review employed a qualitative methodology grounded in thematic analysis to synthesize existing literature on women entrepreneurs driving fintech growth in Sub-Saharan Africa as shown in Table 1. Data were collected through a systematic review of peer-reviewed journal articles, institutional reports, policy briefs, and grey literature published between 2015 and 2025. Online academic databases such as Scopus, Google Scholar, and Web of Science provided access to relevant studies ( Martín-Martín et al, 2021).

**Table 1.** Key strategies empowering women entrepreneurs in fintech business growth across sub-saharan Africa.

Strategy	Main technologies/tools	Key benefits for women entrepreneurs	Country examples	Supporting policies
**Leveraging Mobile Technology**	Mobile phones, mobile money platforms (e.g., M-Pesa, Numida, Paga)	Expands financial access, facilitates microloans and payments, enhances market reach	Kenya (M-Pesa), Uganda (Numida), Nigeria (Paga)	Nigeria’s Financial Inclusion Strategy
**Utilizing Artificial Intelligence (AI)**	AI tools for customer profiling, risk assessment, and predictive analytics	Enables alternative credit scoring, personalized services, expanded customer base	Uganda (Numida), South Africa (JUMO)	AU Digital Transformation Strategy, data governance frameworks
**Adopting Blockchain**	Decentralized ledgers, smart contracts, digital ID verification	Ensures secure, transparent transactions, builds trust, supports cross-border trade	Kenya (BitPesa), Nigeria (Kora), Ghana (AgroCenta)	AfCFTA Protocol on Digital Trade, blockchain regulation
**Adopting Blockchain**	Decentralized ledgers, smart contracts, digital ID verification	Ensures secure, transparent transactions, builds trust, supports cross-border trade	Kenya (BitPesa), Nigeria (Kora)	AfCFTA Protocol on Digital Trade, blockchain regulation
**Harnessing Digital Payments & E-commerce**	Mobile wallets, QR codes, contactless payments, online stores	Boosts sales, supports formalization, opens regional/international markets	Nigeria (Flutterwave), Kenya (Pesapal), South Africa (Yoco)	Kenya’s Digital Economy Blueprint, interoperability policies
**Collaborating with Innovation Hubs**	Incubators, accelerators, mentorship, seed funding	Enhances skills, fosters innovation, supports business model growth	Ghana (MEST), Kenya (iHub/AkiraChix)	AU Digital Transformation Strategy, women-focused innovation policies

### Search keywords

The search strategy included a combination of keywords and Boolean operators to maximize retrieval of relevant literature ( Wang et al., 2023, July; Chigbu et al., 2023). Keywords included: “women entrepreneurs AND fintech,” “mobile technology AND Sub-Saharan Africa,” “AI AND risk assessment AND financial inclusion,” “blockchain AND women-led businesses,” “e-commerce integration AND fintech,” and “innovation hubs AND incubators AND women entrepreneurship.”

### Inclusion criteria

Studies were included if they met the following criteria: publication date from 2021 to 2025; focused on Sub-Saharan Africa; provided empirical data or case studies on women entrepreneurs engaging in fintech; addressed at least one of the five thematic areas (mobile technology, AI, blockchain, e-commerce integration, innovation hubs/incubators); and were available in English. Priority was given to sources offering clear connections between fintech innovations and women’s business outcomes.

### Exclusion criteria

Literature was excluded if it did not specifically address women entrepreneurs or fintech within the Sub-Saharan African context. Studies focused exclusively on general entrepreneurship without gender considerations, fintech studies unrelated to the selected themes, and publications lacking empirical or policy-based evidence were also omitted. Non-English publications were excluded due to resource limitations.

### Data analysis

Thematic analysis followed Braun and Clarke’s six-phase framework ( Byrne, 2022): familiarization with data, generating initial codes, searching for themes, reviewing themes, defining/naming themes, and producing the final report. Relevant data were coded inductively and deductively around the five pre-defined thematic areas: Leveraging Mobile Technology for Market Reach; Utilizing AI for Customer Insights and Risk Assessment; Adopting Blockchain for Secure and Transparent Transactions; Harnessing Digital Payment Solutions and E-commerce Integration; and Collaborating with Innovation Hubs and Incubators for Capacity Building. Patterns, contradictions, and emerging trends were identified during coding.

### Evaluation process

To enhance reliability, peer-reviewed articles were prioritized, and triangulation was used by comparing insights across academic sources, institutional reports, and case studies. Thematic saturation was achieved when no new significant patterns emerged from additional literature. Reflexivity was maintained to account for potential researcher biases, and critical appraisal of included literature followed established frameworks for qualitative synthesis ( Flemming & Noyes, 2021).

### Theoretical framework

This review will be anchored on Everett’ Diffusion of Innovations Theory (1962), which posits that innovations spread through a social system over time via a process involving communication channels and the influence of various adopter categories ( Spann et al., 2022). According to the theory, the adoption of new ideas or technologies depends on key attributes such as the relative advantage of the innovation, its compatibility with existing values and needs, its complexity, trialability, and observability ( Okour et al., 2021). These factors determine how quickly and widely an innovation is embraced by individuals or groups.

The theory is highly relevant as a framework to guide women entrepreneurs’ innovation for fintech business growth in Sub-Saharan Africa. It provides a lens to understand how women identify, adopt, and diffuse fintech innovations within their communities and networks. By emphasizing the social and communication processes involved, the theory helps explain how women entrepreneurs can overcome barriers such as limited access to information, gender biases, and lack of technical skills. Furthermore, it highlights the importance of tailoring fintech solutions to align with the specific financial behaviors and needs of underserved populations, thereby increasing adoption rates. Using this framework, the study can explore strategies that support women-led fintech ventures in scaling their innovations, facilitating wider financial inclusion, and driving sustainable business expansion.

### Literature review

Women entrepreneurs in Sub-Saharan Africa play an increasingly significant role in the fintech sector, leveraging innovation to address financial inclusion gaps and drive economic growth. Modern innovations can offer critical opportunities for business scaling, service delivery, and market expansion as explained in Table 2.

**Table 2.** Thematic areas of fintech innovation supporting women’s entrepreneurial participation.

Strategic area	Key enablers & findings	Critical gaps & contradictions	Future research needs
**Leveraging Mobile Technology**	Success of M-Pesa, Numida, Paga; proven financial inclusion benefits	Overgeneralized impacts; ignores rural–urban divides; insufficient data on long-term digital literacy effectiveness	Disaggregated gender data; scalability studies; regulatory reforms
**Artificial Intelligence for Insights & Risk**	Enhances credit profiling & customer segmentation; supports alternative credit scoring	Limited to Anglophone countries; risk of algorithmic bias; inadequate long-term studies	Evaluate algorithmic fairness; empirical studies on sustainability; regulatory sandbox effectiveness
**Blockchain for Secure Transactions**	Improves security, transparency, cross-border payments; supports smart contracts	High digital skills & infrastructure barriers; insufficient gender-focused evidence; policy-practice gaps	Gender-sensitive adoption studies; assess rural women’s uptake; policy harmonization effectiveness
**Digital Payments & E-commerce**	Supports financial formalization, diversified payments (e.g., Flutterwave, Yoco)	Rural digital divide; high internet costs; policy implementation barriers	Evaluate long-term adoption; study ecosystem resilience; address structural socio-economic barriers
**Innovation Hubs & Incubators**	Mentorship, training, funding; fosters skills & growth (e.g., iHub, CcHub)	Limited rural reach; short-term focus; mixed evidence on inclusivity	Assess long-term outcomes; study rural scalability; align policy frameworks with grassroots needs

### Leveraging mobile technology for market reach

Leveraging mobile technology for market reach has created transformative opportunities for women entrepreneurs in Sub-Saharan Africa, driving fintech growth and advancing financial inclusion ( Mhlanga, 2025; Abagen & Ochi, 2024). The region has experienced a surge in mobile phone usage, with mobile devices now serving as the main channel for accessing financial services, particularly in areas with limited banking infrastructure ( Cariolle, 2021). Through mobile money platforms, women entrepreneurs are able to provide tailored financial services such as micro-loans, digital savings, and secure payment systems to unbanked and underserved populations ( Phil-Ugochukwu, 2024; Ndung’u, 2025). These innovations strengthen financial independence for both the entrepreneurs and their customers, generating widespread benefits for local economies.

Notable success stories highlight the effectiveness of mobile technology in empowering women entrepreneurs. In Kenya, M-Pesa has transformed financial transactions by enabling users to send, receive, and store money via mobile phones ( Mulili, 2022). Women entrepreneurs have utilised M-Pesa and its associated microloan service, M-Shwari, to streamline business transactions, expand operations, and increase business efficiency ( Macharia, 2022; Onyango et al., 2021). In Uganda, the mobile application Numida has supported women-led businesses by offering unsecured loans based on mobile transaction histories, helping entrepreneurs overcome traditional credit barriers and scale their enterprises ( Mbowa et al., 2023).

The impact of mobile technology extends across the region, including Nigeria, where fintech platforms like Paga have provided inclusive payment solutions using both smartphones and feature phones ( Jacob & Umohb, 2024; Agbelade, 2023). Many women-led businesses in informal sectors rely on such platforms for secure transactions without the need for conventional banks ( Ojarikre et al., 2024). Policy support has been crucial in fostering this transformation. National strategies, such as Nigeria’s Financial Inclusion Strategy, have prioritized mobile money to reach underserved populations, especially women ( Chukwuma-Eke et al., 2025; Soetan & Mogaji, 2024). To sustain and scale these advancements, investments in digital literacy, affordable mobile data, and interoperable payment systems remain essential.

### Utilizing Artificial Intelligence (AI) for customer insights and risk assessment

Utilizing AI for customer insights and risk assessment offers significant opportunities for women entrepreneurs in the fintech sector across Sub-Saharan Africa ( Ahmed, 2021). AI-powered tools provide advanced customer profiling, real-time behavioral analysis, and predictive modeling, enabling better business decisions ( Ramya et al., 2024). Through machine learning, women-led fintech businesses can segment their customers, understand diverse preferences, and design personalized financial products tailored to client needs ( Omiwale, 2024; Liang et al., 2024). This not only builds stronger relationships with customers but also opens pathways to serve traditionally underserved groups, such as women, rural populations, and informal businesses.

AI is also reshaping credit risk assessment by addressing the challenge of limited formal credit histories among low-income groups and informal businesses in the region ( Omokhoa et al., 2024). Traditional banks often hesitate to lend to these groups, but AI-driven systems use alternative data, like mobile phone activity, digital transaction records, and social connections, to generate reliable credit scores ( Adewale et al., 2025; Avickson & Ogunola, 2024). Women entrepreneurs in fintech use these insights to offer microloans and other financial products to excluded populations, helping expand financial access while managing default risks ( Omowole et al., 2024).

Successful case studies demonstrate AI’s potential in supporting women entrepreneurs. In Uganda, Numida has leveraged AI to provide unsecured credit to micro and small enterprises by analyzing transaction histories from mobile money platforms ( Arinze et al., 2024; Maxamuud et al., 2024). Similarly, in South Africa, JUMO collaborates with banks and telecom providers to deliver small loans and savings products, improving access to working capital for women-led businesses ( Ojo, 2020). Strong policy frameworks, such as the African Union’s Digital Transformation Strategy, emphasize the importance of advanced technologies like AI in fostering inclusive economic growth ( African Union Commission, 2021; Gikunda, 2023). Regulatory sandboxes, data protection policies, and investments in digital infrastructure will be essential to fully unlock AI’s benefits for women entrepreneurs in the fintech sector ( Tsanis et al., 2025).

### Adopting blockchain for secure and transparent transactions

Adopting blockchain technology for secure and transparent transactions offers significant potential for women entrepreneurs in the fintech sector across Sub-Saharan Africa ( Mani & Ngigi, 2024; Evans & Oni, 2022). As a decentralized and tamper-proof ledger, blockchain ensures that financial transactions are securely recorded and easily traceable, reducing fraud and operational inefficiencies ( Ahmed, 2025). For women-led fintech businesses, incorporating blockchain provides advantages such as smart contracts, cross-border payment solutions, and robust digital identity verification ( Liang et al., 2024). These features build trust with clients and investors, promote transparency, and help women entrepreneurs expand into regional and international markets, fostering long-term business growth ( Pritiprada et al., 2024).

Several successful applications highlight how blockchain benefits women entrepreneurs in the region. In Kenya, BitPesa uses blockchain to facilitate fast, low-cost cross-border payments, helping women engaged in import and export settle transactions efficiently with global partners ( Metzger et al., 2023). In Nigeria, fintech platform Kora integrates blockchain to enhance payment processing and identity verification, allowing women entrepreneurs to authenticate clients and suppliers securely while minimizing fraud risks ( Olomukoro, 2023; Jacob & Umohb, 2024).

Policy support is essential for maximizing blockchain’s potential in advancing women-led fintech ventures. The African Continental Free Trade Area (AfCFTA) Protocol on Digital Trade emphasizes the need for secure, interoperable digital systems to facilitate blockchain-driven cross-border transactions ( Oluwaferanmi, 2025). Regulatory clarity, combined with targeted training and capacity-building programs for women entrepreneurs, will be critical to ensuring inclusive access to blockchain innovations. These interventions will help women in fintech harness blockchain’s full benefits, promoting financial inclusion and sustainable business development across the region.

### Harnessing digital payment solutions and e-commerce integration

Harnessing digital payment solutions alongside e-commerce integration creates transformative opportunities for women entrepreneurs in Sub-Saharan Africa by expanding market access, boosting sales, and enhancing financial resilience ( Irene et al., 2025; Cordes & Marinova, 2023). With the rapid growth of online marketplaces across the region, the ability to accept secure, diverse, and seamless payment methods has become essential for sustaining competitive businesses ( Amofah & Chai, 2022). By adopting platforms that integrate mobile money, QR codes, and contactless payment technologies, women-led enterprises increase convenience for customers, drive higher sales, and access both national and cross-border e-commerce opportunities ( Arner et al., 2024). These digital ecosystems help women formalize operations, improve access to credit, and support long-term business sustainability ( Thomas, 2025).

Practical examples across Africa demonstrate the positive impacts of digital payments and e-commerce integration. In Nigeria, fintech platform Flutterwave has enabled women entrepreneurs to scale businesses through its integrated payment infrastructure, supporting mobile money, cards, and bank transfers for both domestic and international customers ( Idowu, 2025). Flutterwave’s online store feature has been particularly impactful for women in sectors like fashion and crafts, helping them bypass logistical challenges tied to traditional retail ( Ahmed et al. 2023). In Kenya, women selling agricultural produce benefit from Pesapal’s digital payment options, offering QR code and mobile wallet payments that simplify transactions for customers and reduce dependence on cash ( Bradford, 2022).

South Africa’s Yoco provides another successful example, supporting women entrepreneurs with affordable point-of-sale (POS) systems integrated with e-commerce capabilities. These systems have enabled small businesses to formalize payment processes, build customer trust, and qualify for merchant credit based on transaction records ( Ntando, 2022). Effective policy frameworks are critical to maximize these opportunities. Strategies like Kenya’s Digital Economy Blueprint emphasize the importance of supportive infrastructure, affordable internet, and transparent regulation to foster fintech and e-commerce expansion ( Mugambi & Amayo, 2024)). Policies promoting system interoperability, equitable transaction fees, and targeted digital literacy initiatives are essential to ensure women entrepreneurs fully benefit from the evolving digital economy.

### Collaborating with innovation hubs and incubators for capacity building

Collaborating with innovation hubs and incubators is a pivotal strategy for women entrepreneurs in Sub-Saharan Africa aiming to grow their fintech ventures ( Konyango, 2021). These collaborative spaces provide vital resources such as mentorship, technical training, seed funding, and exposure to emerging technologies ( Ravichandran & Dixit, 2024). Through structured programs offered by accelerators and incubators, women entrepreneurs develop practical skills in areas like product development, business model design, and navigating regulatory landscapes ( Kakeesh, 2024). These partnerships help cultivate a culture of innovation, strengthen leadership abilities, and equip women-led fintech enterprises with the necessary tools to compete in both local and international markets ( Omiwale, 2024).

Success stories across Africa highlight the transformative role of innovation hubs in empowering women entrepreneurs. In Ghana, the Meltwater Entrepreneurial School of Technology (MEST) has trained women in areas like coding and business strategy, leading to the creation of fintech startups such as Kudigo, which offers digital financial tools to informal retailers ( Ackah, D. & Boadu, K. O. (2025)).

In Kenya, iHub Nairobi supports young women through coding bootcamps and entrepreneurial guidance. Graduates of these schemes have gone on to establish fintech ventures contributing to local economies ( Konyango, 2021; Okune & Mutuku, 2023). Policy frameworks across Africa increasingly support such efforts, with initiatives like the African Union’s Digital Transformation Strategy (2020–2030) emphasizing the importance of innovation ecosystems, gender-inclusive policies, and regional collaboration. Dedicated funding for women-focused incubators and effective implementation of these policies will be essential to maximize the impact of these capacity-building initiatives for women in fintech ( POLICY, 2024).

## Discussion of findings

### Leveraging mobile technology for market reach

The reviewed literature has been critically analyzed to identify existing gaps, contradictions, and areas requiring further research. Again, a comparative evaluation with previous studies has also been conducted to highlight similarities and explain any differences observed in the findings.

### Leveraging mobile technology for market reach and financial

The reviewed literature effectively highlights the transformative role of mobile technology in enhancing market reach and financial inclusion for women entrepreneurs in Sub-Saharan Africa. However, several gaps and contradictions remain that warrant further research ( Mhlanga, 2025; Abagen & Ochi, 2024). While case studies like M-Pesa, Numida, and Paga demonstrate successful mobile-based financial models ( Macharia, 2022; Onyango et al., 2021; Mbowa et al., 2023; Jacob & Umohb, 2024; Agbelade, 2023), the literature often generalizes success without critically examining variations in impact across socio-economic groups, regions, and education levels among women entrepreneurs. There is limited exploration of structural barriers such as gendered digital divides in smartphone ownership, disparities in mobile internet access between urban and rural areas, and the role of cultural norms in limiting women’s full utilization of mobile fintech tools. Contradictions also exist between policy commitments to financial inclusion and persistent regulatory hurdles, such as high transaction costs and inconsistent interoperability of mobile money platforms across borders. Additionally, while some studies cite digital literacy initiatives ( Cariolle, 2021; Phil-Ugochukwu, 2024; Ndung’u, 2025), there is insufficient empirical data on the long-term effectiveness of these programs in equipping women with the technical skills required for sustained fintech engagement. Further research is necessary to provide disaggregated data on women’s mobile technology usage, assess the scalability of mobile fintech innovations in different contexts, and explore how tailored regulatory reforms can close inclusion gaps for women entrepreneurs in the sector.

A comparative evaluation with previous studies reveals significant alignment in recognizing mobile technology as a critical driver of financial inclusion for women entrepreneurs in Sub-Saharan Africa. Similar to earlier research by Burns (2018) which highlighted M-Pesa’s transformative impact on household financial stability in Kenya, the reviewed literature reaffirms M-Pesa’s continued influence on women’s entrepreneurial success ( Macharia, 2022; Onyango et al., 2021. Additionally, studies by Lal and Sachdev (2015) also emphasized mobile money’s role in bridging financial access gaps, consistent with findings on platforms like Numida and Paga. However, differences emerge in the granularity of analysis. While past studies predominantly focused on general financial inclusion, the current review expands by providing disaggregated, gender-specific examples and business-oriented outcomes, such as agricultural enterprise. Moreover, whereas earlier studies centered primarily on East African contexts, recent findings incorporate examples from Nigeria and Uganda, reflecting a broader regional spread. The integration of alternative credit scoring by Numida, in particular, represents a departure from traditional mobile money models, addressing gaps identified in earlier literature concerning access to credit for women lacking formal banking histories ( Mbowa et al., 2023). These differences highlight a shift from general access narratives toward nuanced evaluations of fintech’s specific benefits for women-led enterprises in diverse socio-economic environments.

### Utilizing Artificial Intelligence (AI) for customer insights and risk assessment

The literature review on leveraging AI for customer insights and risk assessment offers valuable insights but reveals notable gaps and areas requiring further research. While the review effectively illustrates AI’s potential in enhancing customer profiling and credit risk analysis for women entrepreneurs ( Ramya et al., 2024; Omokhoa et al., 2024), it predominantly draws on success stories from a limited number of fintech startups such as Numida and JUMO ( Arinze et al., 2024; Maxamuud et al., 2024; Ojo, 2020).

This creates a geographic concentration, with insufficient exploration of AI adoption across other diverse contexts in Sub-Saharan Africa, particularly Francophone and Lusophone countries where fintech ecosystems remain under-documented. Additionally, while the literature acknowledges AI’s capacity to generate alternative credit scores ( Adewale et al., 2025; Avickson & Ogunola, 2024), it lacks critical engagement with challenges such as algorithmic bias, data privacy risks, and the ethical implications of deploying AI in financially vulnerable communities. Contradictions also emerge regarding the scalability of AI-driven models; while case studies highlight successful small-scale implementations ( Arinze et al., 2024; Maxamuud et al., 2024; Ojo, 2020), broader empirical evidence on the sustainability and long-term socio-economic impacts of AI-driven fintech on women entrepreneurs remains limited. Further research should focus on longitudinal studies to evaluate the durability of AI-based credit models, strategies to mitigate algorithmic discrimination, and assessments of how regulatory sandboxes influence women’s participation in AI-powered fintech ventures across varied socio-economic contexts.

The findings in this literature review align with previous studies that emphasize the transformative potential of AI in enhancing financial inclusion for women entrepreneurs, particularly through alternative credit scoring and customer segmentation ( Gomber et al., 2018). Similar to earlier research by Adams Becker et al. (2017), this review highlights AI’s ability to leverage non-traditional data sources to bridge credit gaps for informal and underserved populations. Both bodies of literature underscore the importance of mobile transaction data and partnerships with mobile network operators in expanding financial access. However, a key difference observed is the greater emphasis in this review on showcasing women-led fintech initiatives, such as Numida and JUMO ( Arinze et al., 2024; Maxamuud et al., 2024; Ojo, 2020), whereas earlier studies predominantly examined AI-driven fintech solutions in broader, gender-neutral terms. Additionally, while past research often focused on technical feasibility and credit scoring accuracy ( Becker et al., 2017), this review integrates policy dimensions such as regulatory sandboxes and data protection frameworks, highlighting a growing recognition of the need for supportive governance structures. Despite these advances, differences persist regarding the scalability of AI applications for women entrepreneurs, with limited longitudinal data available to assess long-term impacts, a gap that earlier studies also noted but did not resolve, indicating a continued need for sustained empirical research.

### Adopting blockchain for secure and transparent transactions

The literature on adopting blockchain for secure and transparent transactions highlights promising developments for women entrepreneurs in Sub-Saharan Africa but reveals notable gaps and areas requiring further exploration. While existing studies, such as those by Ahmed (2025) and Liang et al., (2024), affirm blockchain’s potential in enhancing transaction security and efficiency, much of the current discourse remains concentrated on general fintech applications with limited gender-specific focus. The review emphasizes practical examples like BitPesa and Kora ( Metzger et al., 2023; Olomukoro, 2023; Jacob & Umohb, 2024), yet lacks comprehensive gender-disaggregated data to measure blockchain’s unique impacts on women’s entrepreneurial success. Additionally, while the literature promotes blockchain as a tool for disintermediation and fairness ( Ahmed, 2025), contradictions persist regarding accessibility challenges, particularly the high technological literacy and infrastructure requirements that disproportionately affect women in rural areas ( Debbarma & Chinnadurai, 2023). Furthermore, although regulatory initiatives like AfCFTA’s Protocol on Digital Trade have been acknowledged ( Oluwaferanmi, 2025), there is insufficient analysis of how national policies align with regional frameworks to support women’s participation in blockchain ecosystems. These gaps highlight the need for future research to provide empirical, gender-sensitive evaluations of blockchain adoption outcomes, investigate barriers to technological uptake by women, and assess long-term effects on financial inclusion and poverty reduction.

The current literature on blockchain adoption for secure and transparent transactions among women entrepreneurs in Sub-Saharan Africa aligns with previous studies in highlighting blockchain’s potential to enhance financial inclusion, reduce fraud, and improve operational efficiency through decentralized and immutable ledgers ( Ohnesorge, 2018). Similar to earlier research emphasizing blockchain’s role in lowering transaction costs and enabling cross-border payments ( Celestin & Vanitha, 2015), this review reaffirms the significance of platforms like BitPesa and Kora in facilitating access to financial services for women-led businesses ( Metzger et al., 2023; Olomukoro, 2023; Jacob & Umohb, 2024). However, unlike some earlier studies that broadly focus on fintech innovations without a gender lens, this analysis specifically underscores women’s empowerment through blockchain-enabled digital identity verification and smart contracts, illustrating sector-specific applications in agriculture and trade. Differences also emerge regarding the extent of policy support; while previous work acknowledges regulatory challenges, this review places greater emphasis on recent regional frameworks like the AfCFTA Protocol, which actively promote interoperable blockchain systems and capacity building for women entrepreneurs ( Oluwaferanmi, 2025). These variations reflect an evolving policy environment and increased recognition of gender-inclusive digital finance, suggesting progress but also the need for continued gender-responsive research and policy development.

### Harnessing digital payment solutions and e-commerce integration

The literature on harnessing digital payment solutions and e-commerce integration for women entrepreneurs in Sub-Saharan Africa highlights substantial progress in improving market access and financial inclusion through platforms like Flutterwave, Pesapal, and Yoco, which facilitate diversified and convenient payment methods (( Idowu, 2025; Bradford, 2022; Ntando, 2022). However, significant gaps remain, particularly concerning the uneven digital infrastructure across rural and underserved areas, which may limit the scalability and equitable reach of these technologies. While the review emphasizes the benefits of formalizing payment systems to enhance access to credit, it largely overlooks challenges related to digital literacy, cybersecurity risks, and cultural barriers that disproportionately affect women entrepreneurs. Contradictions arise in the extent to which policy frameworks effectively address these issues; for instance, although Kenya’s Digital Economy Blueprint promotes digital inclusion ( Mwaniki et al., 2017; Mugambi & Amayo, 2024), practical implementation barriers such as high internet costs and limited interoperability between regional payment systems persist. Further research is necessary to explore how these structural and socio-economic factors interact to influence women’s sustained adoption of digital payments and e-commerce, and to evaluate the long-term impact of integrated digital financial ecosystems on women’s business resilience and growth across diverse Sub-Saharan contexts.

The current analysis of digital payment solutions and e-commerce integration for women entrepreneurs in Sub-Saharan Africa aligns with previous studies that underscore the transformative potential of fintech platforms like Flutterwave and Yoco in enhancing market access, formalization, and financial inclusion ( Ntando, 2022; Idowu, 2025). Similar to earlier research, this review highlights the critical role of mobile money and diversified payment methods in overcoming traditional barriers such as cash dependency and limited banking infrastructure ( van Zanden, 2023). However, while prior studies often emphasized urban-centric benefits and larger fintech ecosystems ( Swain & Das, 2018), the present findings offer more nuanced insights into rural agricultural sectors and small-scale artisanal businesses, expanding the understanding of digital payments’ reach. Differences also emerge regarding the effectiveness of policy interventions; earlier research pointed to regulatory fragmentation as a major challenge ( Kernan, 2018), whereas the current review notes recent strides in national frameworks like Kenya’s Digital Economy Blueprint ( Mugambi & Amayo, 2024), suggesting incremental progress in harmonizing digital financial services. Nonetheless, disparities in internet accessibility and digital literacy continue to moderate these benefits, indicating persistent structural challenges consistent across studies that warrant further investigation.

### Collaborating with innovation hubs and incubators for capacity building

The literature review effectively highlights the significant role of innovation hubs and incubators in building the capacity of women entrepreneurs in Sub-Saharan Africa’s fintech sector, emphasizing mentorship, technical training, and access to funding as key enablers of growth ( Konyango, 2021; Ravichandran & Dixit, 2024; Kakeesh, 2024). However, gaps emerge regarding the scalability and long-term sustainability of these interventions, with limited empirical evidence on the measurable impact of such collaborations on business performance and market expansion beyond initial incubation phases. Contradictions also arise in the literature concerning the inclusivity of these hubs; while some studies portray them as accessible platforms for women ( Omiwale, 2024), others suggest persistent barriers related to geographic location, socio-economic status, and digital literacy that may limit participation for rural or marginalized women entrepreneurs ( Ramasamy et al., 2025; Dua & Yadav, 2025). Furthermore, although policy frameworks such as the African Union’s Digital Transformation Strategy advocate for gender-inclusive innovation ecosystems ( POLICY, 2024), there is scant research evaluating the effectiveness of these policies in practice or how they translate into tangible support at grassroots levels. Additional research is required to explore how innovation hubs can better address intersectional challenges faced by women entrepreneurs, integrate localized needs, and foster sustainable ecosystems that extend beyond urban centers to include underrepresented groups across the region.

The findings presented align with previous studies that underscore the crucial role of innovation hubs and incubators in empowering women entrepreneurs by providing mentorship, technical training, and access to funding, thereby fostering skills development and business growth in the fintech sector across Sub-Saharan Africa ( Nicolopoulou et al., 2017; Gavara & Zarco, 2015). Similar to earlier research, this review highlights success stories like Ghana’s MEST and Kenya’s iHub, which demonstrate how targeted support can address gender-specific financial inclusion gaps and enhance entrepreneurial capacities ( Konyango, 2021; Okune & Mutuku, 2023; Ackah & Boadu, 2025). However, some differences emerge regarding the scope of impact; while earlier studies often focus on urban-centered hubs with relatively limited outreach, the current literature suggests an expanding regional collaboration and more deliberate policy emphasis on gender-inclusive digital ecosystems, as seen in the African Union’s Digital Transformation Strategy ( POLICY, 2024). This policy-driven approach marks a shift toward systemic support beyond individual hubs, aiming to institutionalize gender equity in innovation ecosystems. Nonetheless, variations persist in the degree of policy implementation effectiveness and the inclusivity of incubator programs, with some studies reporting ongoing challenges related to access for women in rural areas and those with lower digital literacy. These differences suggest a growing recognition of structural barriers alongside a more coordinated regional strategy, highlighting the need for continued research on how to translate policy frameworks into practical, scalable interventions that reach diverse groups of women entrepreneurs across Sub-Saharan Africa.

### Theoretical implications/contributions

The study examines relationship between literature review and the underpinning theoretical framework thereby identifying the persistent challenges and eminent gaps as reflected in Table 3.

**Table 3.** Theoretical implications of fintech innovations for women entrepreneurs in sub-saharan Africa: A diffusion of innovations theory perspective.

Theme	Key diffusion attributes	Persistent challenges	Identified gaps/Future research
**Mobile Technology Adoption**	Relative advantage, compatibility, trialability, social systems	Digital literacy gaps, gender bias, poor infrastructure	Need targeted strategies to enhance observability and address structural barriers
**Utilizing AI**	Relative advantage, compatibility, complexity, trialability, observability	Complexity, low digital literacy, lack of gender-sensitive training	Integrate policy support and build capacity to accelerate responsible AI adoption
**Blockchain Adoption**	Relative advantage, compatibility, observability	Technology complexity, digital literacy, policy uncertainty	Expand supportive ecosystems, improve knowledge transfer, and build gender-sensitive training
**Digital Payments & E-commerce**	Relative advantage, compatibility, observability, trialability	Digital literacy gaps, infrastructural limits, gender bias	Improve policy enforcement and expand gender-focused capacity-building for inclusion
**Collaboration with Innovation Hubs & Incubators**	Trialability, observability, relative advantage, social networks	Limited rural access, gender programme gaps, funding issues	Broaden inclusive policy and expand equitable access to innovation support structures

### Aligning leveraging of mobile technology adoption with diffusion of innovations theory

The literature review on leveraging mobile technology for market reach among women entrepreneurs in Sub-Saharan Africa aligns well with Everett Rogers’ Diffusion of Innovations Theory by illustrating how key attributes ( Nkonoki, 2025; Tijani, 2023), such as relative advantage (such as, increased financial access through mobile money), compatibility (tailored solutions like M-Pesa and Numida fitting local financial behaviors), and trialability (mobile platforms allowing easy testing), facilitate the adoption and diffusion of fintech innovations. The review highlights the crucial role of social networks and communication channels in spreading these technologies ( Cariolle, 2021), reflecting the theory’s emphasis on social systems in innovation uptake ( Okour et al., 2021; Kuo et al., 2021). However, persistent challenges remain, including digital literacy gaps, gender biases limiting women’s access to information and technology, and infrastructural barriers such as inconsistent mobile data affordability and coverage. These challenges reveal gaps in current interventions, where diffusion is uneven and often excludes rural or less-educated women, pointing to the need for more targeted strategies to enhance observability and simplify complexity for broader inclusion. Additionally, while the theory explains adoption dynamics ( Menzli et al., 2022), the literature suggests that structural and policy constraints require greater focus, indicating that future research should integrate sociocultural and institutional dimensions alongside diffusion theory to better address systemic obstacles impeding women entrepreneurs’ full participation in fintech innovation ecosystems.

### Aligning utilization of AI with diffusion of innovations theory

The literature on utilizing AI for customer insights and risk assessment in women-led fintech ventures in Sub-Saharan Africa aligns closely with Everett Rogers’ Diffusion of Innovations Theory, particularly regarding the attributes influencing technology adoption such as relative advantage, compatibility, complexity, trialability, and observability ( Anabtawi et al., 2024). The reviewed studies highlight how AI’s relative advantage, through improved customer profiling and alternative credit scoring, facilitates adoption by addressing critical barriers faced by women entrepreneurs, including limited access to formal credit and tailored financial services ( Ramya et al., 2024; Omiwale, 2024; Liang et al., 2024). However, persistent challenges remain, notably the complexity of AI technologies and the limited digital literacy among some women entrepreneurs, which may slow diffusion despite the clear benefits. Furthermore, while the theory underscores the importance of communication channels and social systems in spreading innovations ( Danson, 2025; Wiratomo & Suyitno, 2025), the literature reveals gaps in supportive ecosystems, such as insufficient gender-sensitive training, regulatory constraints, and uneven infrastructure across the region. These gaps suggest that beyond technological innovation, strategic efforts are needed to enhance trialability and observability through targeted capacity-building and policy interventions to accelerate responsible AI adoption among women in fintech.

### Aligning adoption of blockchain with diffusion of innovations theory

The literature on blockchain adoption for secure and transparent transactions among women entrepreneurs in Sub-Saharan Africa aligns with Rogers’ Diffusion of Innovations Theory by illustrating how the relative advantage and compatibility of blockchain technology foster its uptake in fintech ventures ( Adu, 2024; Nwosu, 2021). Case studies like BitPesa demonstrate blockchain’s capacity to address specific needs such as cross-border payments, fraud reduction, and fair pricing, supporting the theory’s emphasis on innovation attributes that drive adoption ( Metzger et al., 2023). However, persistent challenges such as the technology’s complexity, limited digital literacy, and infrastructural constraints reflect barriers that slow diffusion despite perceived benefits. While the theory highlights the importance of communication channels and social systems in innovation spread ( Soare et al., 2022), the literature identifies gaps in effective knowledge transfer and gender-sensitive capacity building that hinder widespread adoption. Regulatory uncertainty and insufficient policy frameworks further limit trialability and observability, suggesting that beyond technological advantages, supportive ecosystems and targeted interventions remain essential to enhance blockchain adoption among women-led fintech businesses in the region.

### Aligning digital payment solutions and e-commerce with innovation diffusion theory

The literature on digital payment solutions and e-commerce integration for women entrepreneurs in Sub-Saharan Africa reflects key principles of Rogers’ Diffusion of Innovations Theory, particularly regarding the relative advantage and compatibility of these technologies with existing business needs ( Awe, 2021; Tchasso, 2024). Platforms like Flutterwave, Pesapal, and Yoco exemplify innovations that offer clear benefits by enhancing convenience, market reach, and financial formalisation, which aligns with the theory’s emphasis on observable and trialable innovations driving adoption ( Idowu, 2025; Bradford, 2022; Ntando, 2022). However, persistent challenges such as digital literacy gaps, infrastructural limitations, and gender biases create barriers to widespread diffusion, underscoring the theory’s recognition of complexity as a deterrent. The literature reveals gaps in addressing these socio-technical obstacles, including insufficient tailored training and limited access to affordable, interoperable payment systems for women entrepreneurs. Additionally, while policy frameworks exist, inconsistent implementation and lack of gender-focused support inhibit the scaling and sustainability of fintech innovations. Thus, the relationship between the literature and the theoretical framework highlights the need for integrated approaches that combine technological advantages with targeted social and policy interventions to overcome persistent barriers and promote inclusive digital financial ecosystems.

### Aligning collaboration of innovation hubs and incubators with diffusion of innovations theory

The literature on collaboration with innovation hubs and incubators closely aligns with Rogers’ Diffusion of Innovations Theory by highlighting how structured support systems enhance the trialability, observability, and relative advantage of fintech innovations for women entrepreneurs ( Nkonoki, 2025; Kamel, 2017). Innovation hubs like MEST and iHub provide vital communication channels and social networks that facilitate knowledge sharing, skill development, and mentorship, which are critical in overcoming the complexity and compatibility barriers identified by the theory ( Ackah & Boadu, 2025; Konyango, 2021; Okune & Mutuku, 2023). However, persistent challenges such as limited access to these hubs for rural or less connected women, uneven availability of gender-sensitive programs, and insufficient long-term funding reveal gaps in ensuring equitable diffusion of fintech innovations. Moreover, the literature indicates a lack of comprehensive strategies addressing deep-rooted gender biases and infrastructural inequalities, which impede the scalability and sustained adoption of fintech solutions by women-led ventures. Thus, while the theoretical framework underscores the importance of social processes and tailored innovations, the literature exposes a need for more inclusive policy implementation and expanded capacity-building efforts to bridge these gaps and promote widespread innovation diffusion among women entrepreneurs in Sub-Saharan Africa.

### Recommendations for future research

Future research should explore the nuanced socio-cultural and economic factors that influence women entrepreneurs’ adoption of fintech innovations across diverse Sub-Saharan African contexts, providing deeper insights into region-specific barriers and enablers. Longitudinal studies are needed to assess the sustained impact of blockchain, digital payment solutions, and innovation hub collaborations on women-led fintech ventures’ growth and financial inclusion over time. Investigating the effectiveness of policy interventions and regulatory frameworks in promoting equitable access to fintech resources for women will offer valuable guidance for policymakers. Additionally, research could examine how emerging technologies such as artificial intelligence and decentralized finance (DeFi) can be tailored to address women entrepreneurs’ unique challenges. Finally, comparative studies between urban and rural women entrepreneurs would enrich understanding of digital divide issues and inform targeted strategies for inclusive fintech development.

## Conclusion

This review has demonstrated that empowering women entrepreneurs in Sub-Saharan Africa through fintech innovation is both a practical necessity and a strategic opportunity for inclusive economic growth. Leveraging mobile technology has emerged as a critical tool for enhancing market reach, particularly in overcoming geographical and infrastructural limitations that often constrain women-led businesses. By utilizing AI for customer insights and risk assessment, women entrepreneurs can make data-driven decisions, optimize service delivery, and reduce exposure to financial risks, thereby increasing competitiveness in a dynamic fintech ecosystem. The adoption of blockchain technology further strengthens business transparency, security, and trust, providing women with secure platforms for cross-border transactions and improved financial credibility. Integration of digital payment solutions with e-commerce platforms has expanded women’s access to both local and global markets, while simultaneously improving operational efficiency and access to credit through verifiable transaction records. Finally, collaboration with innovation hubs and incubators has proven instrumental in providing women with the technical skills, mentorship, and networks necessary for scaling fintech enterprises and navigating complex regulatory environments. Despite these advancements, persistent challenges such as digital literacy gaps, gender biases, and inconsistent policy implementation continue to hinder the full realization of fintech’s potential for women entrepreneurs. Addressing these gaps through targeted policies, sustained capacity building, and context-specific innovations will be critical in fostering sustainable fintech-driven growth for women across Sub-Saharan Africa.

### Recommendations to address fintech business growth

Concrete policy recommendations aligned with the five thematic areas reviewed should focus on fostering an enabling ecosystem that addresses systemic gaps and accelerates women-led fintech growth. To leverage mobile technology for market reach, governments and private sector actors should prioritize expanding mobile network coverage and reducing data costs, particularly in underserved regions, ensuring women entrepreneurs can fully utilize mobile platforms for business scalability, as demonstrated by the success of M-Pesa in Kenya. For utilizing artificial intelligence (AI), policymakers should incentivize AI-driven fintech innovations through research grants and promote ethical data policies that safeguard women’s data privacy while enabling access to advanced analytics for customer engagement and credit assessment, similar to innovations led by firms like Jumo in South Africa. To advance blockchain adoption, regulatory bodies must develop clear legal frameworks for digital assets and transactions, promoting transparency and security, as seen in the impact of BitPesa (now AZA Finance) in facilitating secure cross-border payments for SMEs. On digital payment and e-commerce integration, policies should enforce interoperability among payment providers, cap transaction fees, and promote access to merchant credit for women, drawing from models like Flutterwave’s ecosystem supporting women-led online businesses across Nigeria. Finally, for effective collaboration with innovation hubs and incubators, governments should allocate targeted funding for women-focused incubation programs and incentivize partnerships between hubs and fintech firms, replicating successes such as the mentorship-driven model at CcHub and the entrepreneurial training at MEST Ghana. These aligned strategies will address persistent digital, financial, and gender gaps, positioning women entrepreneurs as central drivers of inclusive fintech growth in Sub-Saharan Africa.

### Limitations of the study

Major limitations of this study include the limited availability of gender-disaggregated data specific to women-led fintech ventures across Sub-Saharan Africa and the predominantly descriptive nature of existing literature. The scarcity of comprehensive datasets makes it challenging to provide nuanced, region-wide analyses of the impact of fintech innovations on women entrepreneurs, potentially limiting the generalizability of findings. Additionally, much of the reviewed literature relies on case studies and qualitative insights without sufficient longitudinal or comparative analyses, restricting deeper exploration of causal relationships between specific fintech interventions and sustained business growth for women entrepreneurs in the region.

## Data Availability

No data are associated with this article.
