# Supplementary material for: Women Entrepreneurs and Innovation Strategies: Driving Inclusive Fintech Business Growth in Sub-Saharan Africa
Source: F1000Res. 2025 Dec 23;14:822. Originally published 2025 Aug 26. [Version 2] doi: 10.12688/f1000research.167438.2 (PMC12816952; doi:10.12688/f1000research.167438.2)
Supplement: Peer review history

# Reviewer report: Reviewer response for version 2

**DOI:** 10.5256/f1000research.192904.r445215

**Published:** 2026-01-19

**Authors:** Tahir Iqbal

**Affiliations:** 1 University of Zaragoza, Zaragoza, Aragon, Spain

This manuscript makes a valuable and timely contribution to the literature on women’s entrepreneurship and inclusive fintech innovation in Sub-Saharan Africa. The study is well grounded in relevant theory, demonstrates methodological transparency, and offers clear policy relevance. The thematic analysis is coherent and well supported by current literature. Overall, the article is rigorously developed and suitable for indexing.

Is the review written in accessible language?

Yes

Are all factual statements correct and adequately supported by citations?

Yes

Are the conclusions drawn appropriate in the context of the current research literature?

Yes

Is the topic of the review discussed comprehensively in the context of the current literature?

Yes

Reviewer Expertise:

My current research has a strong interdisciplinary focus, combining the perspectives of management science, entrepreneurship, green leadership, firm performance, digital transformation, and Sustainability.

I confirm that I have read this submission and believe that I have an appropriate level of expertise to confirm that it is of an acceptable scientific standard.

# Reviewer report: Reviewer response for version 1

**DOI:** 10.5256/f1000research.184554.r433537

**Published:** 2025-12-01

**Authors:** Saurabh Chandra

**Affiliations:** 1 Bennett University, Noida, Uttar Pradesh, India

The review employed in the paper which is  qualitative methodology grounded in thematic analysis to synthesize existing literature on women entrepreneurs driving fintech growth in Sub-Saharan Africa.

 This qualitative review could have tried to strengthen the coherence between the sections of the paper.

 The recommendation could have been pragmatic with reference to the changes in the Policy framework.

Is the review written in accessible language?

Yes

Are all factual statements correct and adequately supported by citations?

Yes

Are the conclusions drawn appropriate in the context of the current research literature?

Yes

Is the topic of the review discussed comprehensively in the context of the current literature?

Yes

Reviewer Expertise:

Law and Policy.

I confirm that I have read this submission and believe that I have an appropriate level of expertise to confirm that it is of an acceptable scientific standard.

# Response

**Published:** 2025-12-01

**Authors:** tom ongesa

I am in agreement with your comments. Thank you so much for your time to read the manuscript.

# Reviewer report: Reviewer response for version 1

**DOI:** 10.5256/f1000research.184554.r422275

**Published:** 2025-11-10

**Authors:** Elona N Ndlovu

**Affiliations:** 1 BizPreneur, Pretoria, South Africa

This narrative literature review examines the ways in which women entrepreneurs in Sub-Saharan Africa are utilising fintech innovations, such as mobile money, artificial intelligence, blockchain, digital payment systems, and collaboration with innovation hubs, to promote inclusive business development and enhance financial inclusion. Employing a qualitative, thematic analysis framework, the study synthesises findings from peer-reviewed literature and grey sources (2015–2025), grounded in Everett Rogers’ Diffusion of Innovations Theory.

 The article identifies opportunities and barriers women face in fintech ecosystems and offers policy recommendations for inclusive, gender-responsive fintech development. Five core thematic areas structure the analysis: mobile technology, AI, blockchain, digital payments and e-commerce, and innovation hubs/incubators.

**Area**

**Recommendation**

**Theoretical Development**

 Extend the application of Diffusion of Innovations by proposing a new model or framework that reflects the gendered, digital and informal economy intersection.

**Policy Relevance**

 Strengthen and structure the recommendations using SMART criteria (Specific, Measurable, Achievable, Relevant, Time-bound). Link each thematic area to a clear policy actor and mechanism.

**Comparative Insight**

 Consider deepening contrast across Anglophone vs Francophone countries, rural vs urban areas, or youth vs older women to highlight policy nuance.

**Rigour Enhancement**

 Explicitly report on the search and screening process, quality assessment of included studies, and reflexivity in analysis. This is vital for trustworthiness.

**Visual Data**

 Include figures or conceptual diagrams to summarise findings or propose models: the current tables lack analytical richness.

**Editorial Quality**

 Tighten language, remove redundancy, and enhance clarity of transitions between thematic areas. Also, avoid repetition of content already covered in previous sections.

 The article is promising and academically relevant, and the conclusions are reasonable given the evidence reviewed. However, methodological steps must be made more transparent, and analytical rigour strengthened, before the manuscript can be regarded as scientifically sound.

 Therefore, the most appropriate classification at this stage is:

**Approved with Reservations**

Is the review written in accessible language?

Yes

Are all factual statements correct and adequately supported by citations?

Yes

Are the conclusions drawn appropriate in the context of the current research literature?

Yes

Is the topic of the review discussed comprehensively in the context of the current literature?

Yes

Reviewer Expertise:

Women Entrepreneurship, Women Entrepreneurship and Financial Inclusion, Entrepreneurship Education, Entrepreneurial Coaching, Coaching

I confirm that I have read this submission and believe that I have an appropriate level of expertise to confirm that it is of an acceptable scientific standard, however I have significant reservations, as outlined above.
